# Low-dose prednisone is an effective rescue for deteriorating semen parameters following vasovasostomy

**DOI:** 10.1590/S1677-5538.IBJU.2023.0402

**Published:** 2024-03-18

**Authors:** Joshua White, Katherine Campbell, Nicholas Deebel, Akhil Muthigi, Francesco Costantini Mesquita, Lucas Campos, Christabel Egemba, Maria Camila Suarez Albaraez, Braian Ledesma, Jesse Ory, Ranjith Ramasamy

**Affiliations:** 1 University of Miami Miller School of Medicine Department of Urology Miami Florida United States of America Department of Urology, University of Miami Miller School of Medicine, Miami, Florida, United States of America; 2 Wake Forest University Department of Urology Winston-Salem North Carolina United States of America Department of Urology, Wake Forest University, Winston-Salem, North Carolina, United States of America; 3 Universidade Federal de Minas Gerais Department of Urology Belo Horizonte MG Brasil Department of Urology, Universidade Federal de Minas Gerais - UFMG, Belo Horizonte, MG, Brasil; 4 Dalhousie University Department of Urology Halifax NS Canada Department of Urology, Dalhousie University, Halifax, NS, Canada

**Keywords:** Prednisone, Vasovasostomy, Semen

## Abstract

**Objective:**

This retrospective study aimed to evaluate the effectiveness of low-dose prednisone as a rescue therapy for patients with deteriorating semen parameters following vasovasostomy.

**Materials and Methods:**

Electronic medical records were queried at the University of Miami with documented CPT code 55400 (Bilateral Vasovasostomy) between January 2016 and April 2023. Records were then reviewed to identify patients who demonstrated ≥50% decrease in semen parameters, specifically sperm concentration, motility and total motile sperm count. Patients who were treated with 6 weeks of low-dose prednisone were identified, and baseline semen parameters and subsequent changes after prednisone therapy were assessed. A Mann-Whitney U Test was used to compare semen parameter changes before and after prednisone. Adverse effects associated with prednisone were monitored.

**Results:**

A total of 8 patients were identified with deteriorating semen parameters who were treated with 6 weeks of low-dose prednisone. Following prednisone therapy, all patients demonstrated improvements in total motile sperm count (TMSC), with a median improvement of 6 million. The median relative improvement in TMSC was 433%. Sperm concentration and motility also improved compared to post-operative baseline. No adverse effects were reported during the treatment period.

**Conclusions:**

Low-dose prednisone therapy appears to be a safe and effective intervention for managing deteriorating semen parameters following VV. The observed improvements in TMSC suggest the potential of prednisone to rescue patients with delayed failure after VV. Further research with larger sample sizes is warranted to confirm the safety and efficacy of low-dose prednisone as a rescue therapy in this specific patient population. Optimizing VV outcomes is crucial in male infertility, and further exploration of steroid therapy and innovative biotechnologies is warranted.

## INTRODUCTION

Vasovasostomy (VV) and vasoepididymostomy (VE) are reconstructive treatments for men with Obstructive Azoospermia (OA) seeking fertility following vasectomy. While postoperative patency and pregnancy rates vary between series, the overall success for VV procedures is quoted to be high at 75-95% (1-3). Following a VV procedure, serial semen analyses (SAs) are performed to assess for the return of sperm to the ejaculate. Late failure has been defined in a myriad of ways in the literature. For instance, late failure has been defined as regression from either presence of sperm in the ejaculate or the presence of less than 2 million total motile sperm (3-5). Farber et al. systematically examined the kinetics of sperm return to the ejaculate following a Vasectomy Reversal (VR) procedure using a meta-analysis approach. Their work showed that the mean time to patency following a VV procedure ranged from 1.7-4.3 months (7). They also demonstrated that the mean time to late failure (secondary obstructive azoospermia) was approximately 9.7-13.6 months.

Progressive inflammatory scarring of the mucosa is considered a contributing factor to late failure, indicating the need for exploration into anti-inflammatory interventions (3, 6). Although a study by Machen et al. demonstrated the efficacy of a high-dose prednisone regimen, concerns arise regarding the associated immunosuppressive effects (8). We therefore, aimed to perform a retrospective analysis of our management of this scenario to assess whether an alternative low-dose prednisone regimen was effective in rescuing patients with deteriorating semen kinetics following VV.

## MATERIALS AND METHODS

In accordance with ethical guidelines, this study received IRB approval from the University of Miami prior to the commencement of data collection. The study patient cohort was identified by querying electronic medical records at the University of Miami with documented CPT code 55400 (Bilateral VV) between January 2016 and April 2023. Inclusion criteria for consideration included the following: men undergoing bilateral VV per CPT code 55400 patients who demonstrated a ≥ 50% decrease in semen parameters: specifically, sperm concentration, motility, and total motile sperm count (TMSC) on postoperative SA. Patients were also individually contact by telephone to inquire about long term pregnancy outcomes.

Demographic data, prednisone prescription, and SA results (serially, before and after prednisone) were obtained for each patient with the goal of evaluating efficacy of daily 5mg oral prednisone for 6 weeks after demonstration of deteriorating semen parameters after vasectomy reversal. While this was a retrospective analysis, our institutional protocol includes SA checks in the following manner: 2-3 weeks postoperatively, at three months, subsequently every three months. Patients who had a low TMSC on their first SA after surgery and were immediately placed on prednisone were excluded in order to capture only those who initially presented with a normal SA followed by delayed failure. The primary outcome measure was change in TMSC after treatment with prednisone in this specific population. Exclusion criteria included: patients who underwent VE (unilateral or bilateral), perioperative testosterone therapy, patients with postoperative COVID-19 infection, patients with grade II-III varicocele (unilateral or bilateral), history of inguinal hernia repair, or failure to obtain postoperative SA examination. Patients were screened for concomitant selective estrogen modulator (SERM) or gonadotropin therapy during their perioperative course.

To compare changes in semen parameters before and after prednisone treatment, a Mann-Whitney U test was employed due to the non-parametric nature of the data. This statistical analysis was chosen to evaluate the potential impact of prednisone on semen parameters, considering the absence of normal distribution in the dataset.

## RESULTS

There was a total of 8 men identified with deteriorating semen parameters following VV. The median age of these men was 35.5 years (interquartile range (IQR): 33.8-38.3). The median baseline TMSC was 5 million (interquartile range (IQR): 0.41-13.5) (Table-1) at a median of 4.6 months (IQR: 4.0-7.9) from reconstructive surgery. All patients had deteriorating semen parameters in terms of sperm concentration, percent motility, and total motile sperm count. The median time from surgery to prednisone therapy was 9.5 months (IQR: 7.2-13.0) and all patients were treated with 5mg po prednisone for six weeks. No minor or major adverse effects were reported associated with prednisone therapy, and all patients reported completing the entire course of treatment. None of the eight patients were using perioperative SERM or gonadotropin therapy.

**Table 1:** Baseline Cohort Characteristics.

Patient Variables		Overall
Number of patients		8
Age at vasectomy (years)		
	Median (IQR)	34.5 (33.8 - 38.3)
Time from VV to baseline semen analysis (months)		
	Median (IQR)	4.6 (4.0-7.9)
Sperm concentration (x10^6^/cc)		
	Median (IQR)	9(3.4-11.5)
Motility (%)		
	Median (IQR)	29 (7-51)
Total motile sperm count (x10^6^)		
	Median (IQR)	5(0.45-13.5)

**Figure 1:**
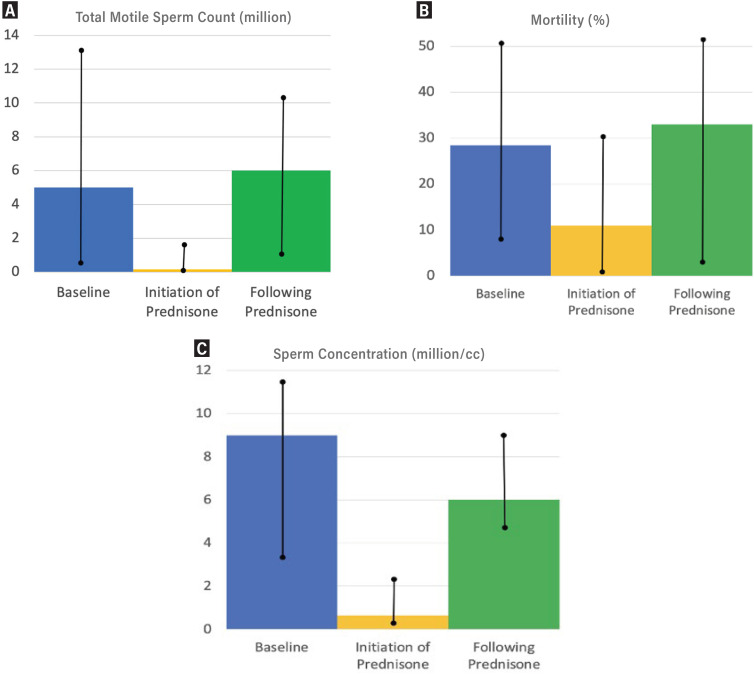
A: Sperm concentration at baseline, before and after prednisone therapy; B: Sperm motility at baseline, before and after prednisone therapy, C: Total motile sperm count at baseline, before and after prednisone therapy.

Following prednisone therapy, the TMSC improved in all patients, with a median improvement of 6 million (IQR: 4.5-9) (Table-2). The median relative improvement in TMSC was 433% (IQR: 241-800). Sperm concentration improved following prednisone therapy but did not return to the baseline concentration (Figure-1A). Both the median motility and TMSC improved when compared to post-operative baseline following prednisone therapy (Figure-1B and C).

**Table 2:** Semen parameters before and after prednisone therapy.

Semen parameter		Prior to Prednisone	Following Prednisone
Sperm concentration (x10^6^/cc)			
	Median (IQR)	0.65(0.18-2.40)	6 (4.5-9)
Motility (%)			
	Median (IQR)	11 (0-29.5)	33 (4-53)
Total motile sperm count (x10^6^)			
	Median (IQR)	0.15(0-1.3)	6(1.1-10.5)

Upon calling patients, one couple had become pregnant through IVF (by choice). Three men had not achieved pregnancy. Of these three, one had chosen to wait to conceive and was actively attempting IUI at the time of statistical analysis and additional two were awaiting further workup of their female partner. Finally, one patient declined further discussion with the team and three patients were unreachable upon multiple attempts.

## COMMENT

The occurrence of deteriorating semen parameters following VV may indicate progressive scarring of the anastomosis (7). To address this issue, we evaluated the efficacy of low-dose prednisone in managing deteriorating semen parameters in the context of delayed failure following VV. Prednisone, a corticosteroid medication known for its anti-inflammatory and immune-modulating properties, has previously been used to mitigate scar formation (9). However, to our knowledge, this is the first study to specifically assess the effectiveness of low-dose prednisone in this particular clinical scenario.

In our study, we employed physiological doses of prednisone, with a daily dosage of 5 mg. This raises the question of why we observed significant improvements in semen parameters. Physiological doses of prednisone have been associated with minimal adverse effects, and side effects such as hyperglycemia, weight gain, hypertension, and edema, primarily occur when administered at high doses or over an extended period (10). We observed no adverse effects in our patient population during the six-week protocol of 5mg daily. This highlights the potential safety and applicability of low-dose prednisone in managing deteriorating semen parameters following VV, especially considering the limited contraindications associated with this treatment (7).

Anastomotic obstruction following VV can occur due to various factors, including perivasal hematoma, anastomotic tension, de-vascularized anastomosis, sperm granuloma, or suture-related inflammatory reactions (7). In our limited series, the use of immunomodulatory therapy, such as prednisone, demonstrated efficacy in managing patients with deteriorating semen parameters. TMSC, which had deteriorated to levels compatible only with IVF for successful pregnancy, significantly improved in all patients after prednisone therapy (Table-3). The post-therapy TMSC reached levels suitable for intrauterine insemination (IUI) and even natural conception (11). The clinical implications of these findings if repeatable may serve to alleviate both financial and psychosocial burdens experienced by couples experiencing infertility (12). However, it should be noted that there is a scarcity of literature evaluating the efficacy of prednisone rescue treatment for deteriorating semen kinetics following VV, highlighting the need for further research in this area.

**Table 3:** Patient profile: prednisone x SA improvements.

Time from VV to prednisone (days)	Pre-prednisone TMSC	Gross increase in TMSC	% increase from previous SA in TMSC
332	9	0.1	1
227	50	8.8	44
375	52	4	4
239	30	1	0.125
430	0	2.7	9
184	0	4.9	49
981	18	3.5	1
156	4	12	6

It is important to acknowledge the limitations of our study. As a retrospective, single-institution study with a small sample size, the potential for selection and ascertainment bias cannot be ruled out. Importantly, this study was not designed as a clinical trial therefore there is no control arm to investigate the natural healing process of a matched cohort of men not receiving prednisone. Though our preliminary results are promising, the optimal dosing, treatment schedule, and patient selection criteria remain unknown. Further work such as a large, collaborative multi-institutional study is necessary to better understand this new treatment option. Additionally, the lack of available pregnancy data limits our ability to assess the direct impact of low-dose prednisone therapy on fertility outcomes. Nevertheless, the well-documented safety profile of low-dose prednisone therapy supports its potential as an effective rescue treatment for deteriorating semen parameters following VV (10).

Our results suggest that modulation of the inflammatory system through the use of prednisone may be beneficial for men experiencing deteriorating semen parameters following VV with presumed anastomotic obstruction. Considering the critical importance of optimizing VV outcomes for male infertility specialists, further investigation into the potential benefits of steroid therapy in this patient population is warranted. Furthermore, exploring innovative biotechnologies designed for wound healing and the reduction of tissue inflammation, such as the BioD™ tri-layer amniotic membrane may offer additional avenues to optimize surgical outcomes in male reproductive medicine (13, 14). Future work is warranted to determine whether anti-inflammatory grafts have a role in the reduction of inflammation and late failure in patients undergoing vasal reconstruction. We hope that further research into the efficacy of prednisone and the application of products designed for wound healing or tissue regeneration may provide benefits to patients with failure after vasectomy reversal.

## CONCLUSIONS

Prednisone therapy appears to be a safe and effective modality for managing deteriorating semen parameters following VV. Low-dose prednisone for six weeks following deterioration of semen parameters resulted in improvements in TMSC. TMSC improved to levels compatible with both IUI or natural conception following prednisone therapy. Our results call for larger confirmatory studies to confirm the safety and efficacy of low-dose prednisone for delayed failure following VV.
